# Climate Change, Migration, and Civil Strife

**DOI:** 10.1007/s40572-020-00291-4

**Published:** 2020-10-13

**Authors:** Satchit Balsari, Caleb Dresser, Jennifer Leaning

**Affiliations:** 1 Department of Emergency Medicine, Harvard Medical School and Beth Israel Deaconess Medical Center, Boston, MA 02215 USA; 2 FXB Center for Health and Human Rights, Harvard University, Boston, MA 02215 USA; 3 Climate and Human Health Fellowship, Emergency Department, Beth Israel Deaconess Medical Center, Boston, MA 02215 USA; 4 Department of Emergency Medicine, Harvard Medical School and Brigham and Women’s Hospital, Boston, MA 02215 USA

**Keywords:** Climate change, Migration, Climate refugees, Adaptation, Civil strife

## Abstract

**Purpose of Review:**

In this article, we examine the intersection of human migration and climate change. Growing evidence that changing environmental and climate conditions are triggers for displacement, whether voluntary or forced, adds a powerful argument for profound anticipatory engagement.

**Recent Findings:**

Climate change is expected to displace vast populations from rural to urban areas, and when life in the urban centers becomes untenable, many will continue their onward migration elsewhere (Wennersten and Robbins 2017; Rigaud et al. 2018). It is now accepted that the changing climate will be a threat multiplier, will exacerbate the need or decision to migrate, and will disproportionately affect large already vulnerable sections of humanity. Worst-case scenario models that assume business-as-usual approaches to climate change predict that nearly one-third of the global population will live in extremely hot (uninhabitable) climates, currently found in less than 1% of the earth’s surface mainly in the Sahara.

**Summary:**

We find that the post–World War II regime designed to receive European migrants has failed to address population movement in the latter half of the twentieth century fueled by economic want, globalization, opening (and then closing) borders, civil strife, and war. Key stakeholders are in favor of using existing instruments to support a series of local, regional, and international arrangements to protect environmental migrants, most of whom will not cross international borders. The proposal for a dedicated UN agency and a new Convention has largely come from academia and NGOs. Migration is now recognized not only as a consequence of instability but as an adaptation strategy to the changing climate. Migration must be anticipated as a certainty, and thereby planned for and supported.

## Introduction

### Trends in human migration

People have always been on the move in search of a better life and often simply for survival. Population movements, both voluntary and involuntary, have been of interest to those in power and especially so in the last 400 years, coinciding with the period of great explorations, wars of extensive scale, accelerated population growth, and increased control of political borders. The international legal regime created in the years after World War II, when at least 50 million refugees were stranded in Europe and uncounted millions rendered homeless in Africa and Asia, established rules and regulations relating to definitions and options for people who had left one country for any reason and desired entry into another. These rules have been used to establish the legitimacy of an individual’s claim to be a refugee. In the decades since the end of WWII, these rules have proved insufficient to capture the needs and motives of desperate people on the move [••^1^, ^2^]. Migration has now become the reality for nearly one billion people. On a seasonal basis, the numbers are larger, although on smaller temporal and spatial scales. In this paper, we use movement and migration interchangeably to mean displacement, whether voluntary or forced, temporary or permanent, and across spatial scales.

Diverse political, economic, and environmental causes have triggered large migrations in the past five decades (see Table 1). A surge in human population creating rising resource demands, an explosion in industry and technology with heavy reliance on fossil fuels, and many different kinds of wars and ethno-political conflicts have brought us to this point: nearly one billion people live away from their places of birth, among whom about 270 million (or 3.5% of the global population) have crossed international borders. Those on the move include people seeking work, collective groups of people fleeing war and oppression, environmental and climate refugees, and many who fit several of these categories [••^1^, ^5^–^7^].

**Table 1:** Notable international migrations in the past five decades (non-exhaustive list), reconstructed from “Migration Waves,” National Geographic, August 2019

The 1970s	Bangladesh to India	Conflict
	Ethiopia to Somalia	Famine and conflict
The 1980s	Afghanistan to Pakistan and Iran	Conflict
	Mozambique to Malawi	Famine and war
The 1990s	Rwanda to Congo	Genocide and war
	Migrants return to Afghanistan	Transient cessation of conflict
	Emigration to the USA	US policy changes
	E. European emigration to Germany	Iron curtain lifted
	Emigration to Spain from Algeria, Mali, Somalia, Equatorial Guinea, Côte d’Ivoire, DRC	Economic growth, labor demand; civil conflict, political dissidence, war
	Immigration from India and China	Economic need + ability
	Emigration to Russia	Oil and gas discovery, Soviet collapse
The 2000s	Bangladesh to the Gulf states	Economic opportunity + remittances
	Immigration from India/China continues	Economic need + ability
	Immigration from Mexico trails off	9/11, US border enforcement, market
The 2010s	Emigration to the UAE	Oil wealth, construction boom
	Syria to Turkey, Lebanon, Germany, Jordan	Conflict
	Myanmar to Bangladesh	Rohingya genocide

Bangladesh, Mexico, China, India, Pakistan, and the Philippines have for the past decade continued to send emigrants elsewhere. The Levant has experienced massive population shifts; the Gulf states, Germany, and the US continue to experience a net influx of immigrants. These population shifts, on the surface, are attributed to political and economic triggers, while extreme climate events and the environment may—to some extent—undergird the sociopolitical upheavals [3, 4]

In the first half of the twentieth century, migration into wealthy countries of the North and West was driven by major wars and the collapse of countries. In the 1990s, however, the influence of environmental degradation and then climate-change-induced distress began to emerge in the scientific and policy discourse [8]. This phenomenon is not new—periodic shifts in environmental conditions or sheer over-population of fragile ecosystems have prompted migration for centuries. Advances in remote-sensing and surveillance capabilities that have tracked population displacements in recent years have generated strong empirical evidence that migration is a key survival mechanism in the face of extreme weather events, including floods and droughts; that most climate-related migration takes place across short distances within countries or across contiguous borders; and that households which lack migration options are inherently more vulnerable and less adaptable to the impacts of climatic variability [9]. The drivers of migration are complex and there is now growing recognition that climate change is a threat multiplier, meaning that while it may not directly cause conflict, it can significantly exacerbate the conditions that lead to conflict, destitution and displacement [10].

Until recently, large migrations usually resulted in the eventual integration of people into the social and economic lives of communities and states in their path. The processes were complicated and much hardship and suffering were often experienced by the first generation who made the move. In recent decades, however, the international and regional responses to migration have become much less welcoming and non-porous, requiring large swathes of people to undertake perilous and often fatal journeys by road or by sea to find a route into some country that might allow them entry. Popular discourse has often reduced the complexity underpinning the decision to migrate to a tussle over resources between those native to the land and those that have arrived from elsewhere.

## Climate Change Hazards: Drought, Heat, Sea-Level Rise, and Storms

Over the course of this century, it is expected that heat and irregular rainfall will render many agricultural lands infertile and contribute to land degradation, the expansion of deserts, food insecurity, and permanent alteration of many regional economic systems [••^11^]. There is an extensive history of how prolonged episodes of severe drought in many regions of the world have caused hardship for pastoralists and farming populations whose lives and livelihoods depend upon periodic rains. In general, several years without rainfall are required before these populations decide they must leave [12]. They sell assets to try to survive until the rains come and then over time their animals, and when fully impoverished, they then abandon home [13, 14]. Where people choose to go depends upon location and custom, and their ability to leave; movements may be to urban areas, coastal regions, or adjacent lands which remain fertile. These paths out inevitably lead to social collisions with other populations, and again, consequences vary considerably. The world community has had more experience with periodic drought in sparsely settled rural areas than in dense cities where, in general, wealth and infrastructure have buffered urban populations from the direct effects of decreased rainfall. With accelerating climate change, however, the intensity and duration of droughts are likely to increase, impacting urban water supply [••^11^]; urban heatwaves will also become more frequent [15].

As greenhouse gas concentrations continue to rise, regional warming will intensify [••^11^, ^16^]; models suggest that alterations in atmospheric dynamics may lead to large changes in precipitation, particularly in subtropical and mid-latitude regions. [17–19]. The 2013 IPCC report forecasts that, by the late twenty-first century, intense heat and dryness will affect countries extending across North Africa into Egypt and Sudan and northeast to Saudi Arabia, the Levant including Syria, Iraq, and Iran [20], to parts of India and China in Asia, and across the Southern United States and Mexico [21–23]. Intense regional heat may render some areas uninhabitable [•^24^]. A 2019 model suggests that business-as-usual approaches to climate mitigation and population growth may expose one in three humans in this belt to high temperatures currently found only on 0.8% of the earth’s surface [25].

These environmental conditions raise fundamental concerns about the viability of agriculture in these important regions, as they are seen now to be forcing millions of farmers, pastoralists, and smallholders into increasingly precarious livelihoods and worsening food insecurity [••^11^, ^26^]. In Iran, models suggest that soil may be increasingly infertile by mid-century, contributing to increased dust storms and falling agricultural output [27–29]. In wealthier settings, such as Australia, high transition costs may occur as commercial farmers seek to control additional resources and invest in more efficient systems to maintain agricultural output [30]. An additional risk factor may be the habitation of regions that will become more desirable as a result of climate change and may become the object of resource competition with adjacent states or populations [31]. Dependence on water from transboundary rivers that upstream political entities may seek to control also creates inherent vulnerability and opportunities for conflict [32].

Other impacts of climate change will impose similar migration choices. Evidence of accelerating sea-level rise due to climate change is now extensive. Mainstream estimates range from 0.29 to 1.1 m by the end of the twenty-first century [33, 34], and higher rates are possible if ice sheet and permafrost melting occur faster than anticipated [35]. Displacement of populations whose dwellings are inundated is the most obvious concern and is already occurring in some low-lying islands and river deltas. Displacement from these areas will be a widespread issue within a few decades [36–38]. In addition, repetitive instances of saltwater intrusion from sea-level rise and storm surges will render well water non-potable and farmland infertile, causing livelihoods stress even for people whose dwellings are not directly affected by rising waters [39–41].

Storms will increase in severity and frequency as a result of climate change [42–44]. High winds, hail, and heavy rainfall can directly damage crops and can lead to extensive surface flooding, landslides, and structural damage; in the case of tropical cyclones, these may also be accompanied by deadly coastal storm surges causing millions of dollars in damage [45–47]. The resulting physical and emotional trauma, in addition to damage to individual livelihoods, businesses, and entire sectors of the economy can be substantial and, in some cases, accelerate emigration of affected persons or even lead to abandonment of heavily damaged locations [48–50].

In the Arctic, melting permafrost and sea ice are fundamentally altering transportation systems and rendering some subsistence-based lifestyles unsustainable, as maritime resources are harmed by warming, acidifying oceans [33, 51–54]. Wildfire has become a new hazard in the region, threatening settlements in the tundra and boreal forest and in some cases permanently altering the ecosystem [55]. This threat is not limited to the far north; inhabitants of some regions in California may soon be unable to purchase fire insurance, suggesting significant actuarial concern about the long-term habitability of certain fire-prone locations [56].

Current economic and political systems ensure that these slow-moving but inevitable changes will force millions to move for reasons not envisioned in the legal regimes instituted in the 1950s and 60s [57]. How migrants are received in host communities however has profound and long-lasting implications on the future of both. Migration affects most aspects of human social interaction and in itself imposes environmental change when large influxes of refugees lead to increased demands on ecosystem services as well as on space, food, and shelter [58]. In the face of inevitable large-scale migration expected within and from the weak economies of Latin America, Africa, the Middle East, and South Asia, the current xenophobic approach to keeping out migrants ensures devastating and destabilizing consequences for humans and the planet.

## Who Is at Risk?

A full review of specific vulnerable populations in the face of climate change projections has been extensively explored by other authors [59, 60]. Here, we focus on four regions at the confluence of climate-related hazards noted above: the African Sahel, the Middle East and North Africa (MENA), the “Dry Corridor” in Central America, and South Asia, as together they host the world’s most vulnerable billions.

1. The Sahel: Migration and conflict related to climate change and resulting agricultural and ecological problems are increasingly prominent issues in the Sahel, a semi-arid region stretching across Africa from Ethiopia to Senegal. Smallholder rainfed agriculture and herding are becoming increasingly difficult due to unpredictable availability of water and in some locations expansion of the Sahara Desert [23, 61, 62]. These stressors and competition for available resources may have contributed to conflicts in Nigeria [63], Uganda [64], Sudan [65], and Kenya [66], although the relative causal contributions of climate change, governance, population pressures, and preexisting sectarian divisions to outbreaks of organized violence remain a subject of intense debate [31, 67, 68]. Many people facing unpredictable weather, food insecurity, and in some cases violence have left their land for urban centers in African nations, where conflict with existing populations and discord over space and essentials may occur [69–71]. Others have followed longer, more dangerous migration routes across the Sahara and the Mediterranean in hopes of reaching Europe. These routes are characterized by conflict with populations and governments along migration routes and substantial hazards during transport across desert and ocean [66, 72, 73]. Even after arrival in host countries, migrants remain a source of intense political tension and experience a variety of forms of discrimination and violence [74, 75].

2. The MENA Region: Climate models for the RCP8.5 (business-as-usual emissions trajectory) in the Middle East and North Africa estimate that by 2050 there will be at least a 100 days a year (and by 2100, over 200 days) when temperatures will cross into the 90th percentile for the region, with the hottest temperatures exceeding 100°F [20, •^24^]. The same models suggest that in the same time frame (2050) the heat stress on the soil in many areas would desiccate organic life to as much as 8 inches deep, precluding crop growth even if adequate irrigation were provided.
  Although there is some disagreement, the preponderance of the literature suggests that climate change has contributed to civil strife in Syria [76–78]. There, a prolonged drought in the east and northeast linked to climate change forced pastoralists and farmers to abandon their land, sell their animals, and trek to the cities in the western region of Syria. In 2010–2011, it is estimated that approximately one million people migrated to these urban centers, including the seat of the Assad government in Damascus [3, 77]. Underlying political grievances in these urban areas, already stretched for resources by 1.5 million Iraqi refugees from previous years, were aggravated by the addition of this economically disenfranchised population. Street protests against the Syrian government in the spring of 2011 led rapidly to an outright civil war [3]. That war, now in its tenth year, has killed approximately 500,000 people, created 5.5 million refugees and at its peak 10–11 million internally displaced persons. The ongoing ramifications of this conflict are undermining stability in much of the Middle East as well as Europe.

3. Central America: In Central America, another pattern of climate change, drought, livelihood collapse, discord, and distress migration leading to conflict has occurred [79]. Since 2009, the “Dry Corridor” in Nicaragua, Honduras, El Salvador, and Guatemala has experienced a series of devastating multi-year droughts [80]. This series is consistent with the predicted effects of climate change in the region, and there is now substantial evidence linking drought and agricultural hardship to the effects of climate change [••^11^, ^59^, ^81^]. According to the Food and Agricultural Organization (FAO) of the United Nations, crop losses in the region from 2006 to 2016 range from 50 to 90% and left 3.5 million people in need of humanitarian assistance [82]. These losses have had a profound effect on the viability of agricultural livelihoods, with the majority of households resorting to crisis coping methods such as selling agricultural tools for food. Even so, as of the last quarter of 2018, more than 25% of the population in the Dry Corridor are without sufficient income to purchase necessary food [13]. Facing uncertainty about future rainfall, many have chosen to leave, with emigration from the region increasing by 500% between 2010 and 2015 [13, 79, 83, •^84^]. The majority of people moved overland along this “dry” corridor to seek better prospects northwards. This trek has resulted in increasing conflict between migrants and authorities in transit nations and along the southern border of the USA. This border, historically the final destination and hopeful push-off point in the USA for many of those attempting to escape the worsening conditions to the south [85], has for the last five years now been effectively closed [86].

4. South Asia: The densely populated Gangetic plains in South Asia also fall in the zone that will experience high temperatures as a result of climate change in the coming decades. A 2017 model suggested that extremes of wet-bulb temperature in South Asia, under the Representative Concentration Pathway 8.5 (RCP 8.5) scenario, are expected to approach or exceed the critical temperature of 35 °C (95 °F), considered an upper limit of human survivability [87]. In addition, Bangladesh will face the consequences of rising sea levels and coastal flooding that is already imperiling millions of coastal dwellers.
  The South Asia region has already experienced severe heat waves in recent years, including the fifth deadliest heat wave in recorded history killing 3500 people in Pakistan in 2015. Per the 2018 World Bank Report, by 2050, South Asia could see 11–22 million climate migrants in scenarios under models of urgent climate mitigation, and up to 35 million under a more pessimistic scenario [9]. The population is highly dependent on the summer monsoons and rainfed agriculture, making it particularly vulnerable to temperature and rainfall changes. The region is likely to have the highest number of food-insecure people by mid-century [88]. The southern Indian highlands between Chennai and Bangalore (containing many densely populated cities), parts of northwest India, and Nepal may see in-migration, while Bangladesh and the Gangetic corridor from Lahore to Delhi will be hotspots for out-migration. Coastal cities such as Mumbai, Dhaka, and Chennai are likely to reel under the burden of continued rural to urban migration, until intensifying heat, and worsening flooding and coastal storm surges make life untenable for most of the millions who live at the economic margins of these megalopolises, compelling them to move again. Models estimate that the number of people trying to live in the flood plains of South Asia may rise from 29 million in 2000 to at least 110 million by 2060, even as climate change renders these regions more hazardous [7].
  Growing religious and ethnic intolerance in South Asia does not bode well for the region when the overwhelming survival need for populations in this century will be more movement not less. Rohingya refugees from Myanmar are trapped in what is now the world’s largest refugee camp in coastal Cox’s Bazar in Bangladesh, where they are denied formal refugee status, and remain stateless, unable to seek formal work, advance educational opportunities, or do much more than barely survive [89]. In neighboring India, the government launched an attack on its minorities by re-defining citizenship, arresting dissenters, and beginning to build detention camps for Muslims and unregistered populations in the northeast of the country [90, 91]; the border with neighboring Bangladesh, a likely source of climate refugees, is now heavily militarized [92]. India is currently the greatest exporter of migrants—nearly 12 million—to the Gulf states, the USA, the UK, and elsewhere.

The experience of populations in the Sahel, MENA, Dry Corridor, and South Asia, where the climatologic roots of their displacement may overlap considerably with conflicts arising from deep-seated political enmities, suggests patterns of mixed climate change and social crises now occurring in many regions. As they seek stability, livelihoods, and food in new locations, the implications of these movements for affected populations and host regions have become a topic of increasing concern in policy circles although few robust response strategies have emerged from these deliberations. These patterns are expected to intensify and occur more frequently as climate change becomes more extreme in the coming decades.

## Receiving Migrants

A vexed question over the last 30 years for social scientists and demographers has been whether uprooted populations contribute to intra or inter-group conflict during migration or in the host country. The expert consensus from the late 1990s through early 2000s was that societies could adapt, migration was not clearly caused by climate change, and there was little to no evidence for the influence of environmental stress (let alone climate change) on the incidence or persistence of armed conflict [93]. Shifts in these arguments began to emerge in the mid-2000s, as the effects of climate change became more marked [94], the number of resource wars increased with surges in forced migration [78], and research and policy analysis contributed to enhanced understanding of the pivotal negative roles played by internal social fragilities and incompetent or malicious state leaders in the setting of external economic and agricultural shocks [95, 96].

At present, available evidence indicates that distress migration from climate-induced environmental crises does sometimes lead to intense social conflict and in certain instances contributes directly to armed conflict. There are however numerous mediating variables: the capacity of the host government and its economy to absorb the incoming population, the extent to which the incoming population is related politically or socially to another population threatening the state’s internal order, and the underlying historical vulnerability of this society to civil war or armed conflict all play a role [97–99]. These variables are crucial in all assessments of these inter-relationships and the most pivotal of these is the fragility of the government and its recent conflict history. To put the empirical findings positively, a flexible and robust system of governance with a strong economy can respond to an influx of large numbers of people without deep social distress or conflict—provided sufficient foresight and resources are devoted to advance planning and mitigation.

For decades, however, migration was not high on the global political agenda, and not imagined beyond the contours of movements of political refugees. The Millennial Developmental Goals do not pay particular attention to migrants. This stance has changed rapidly in the first ten years of the twenty-first century, as evidence as well as the experience has accumulated to focus attention on the increasing numbers and kinds of distressed migrants moving within countries, within regions, and to Europe and the USA. Some are fleeing extreme weather events or slow-onset but cumulatively dramatic environmental change. Some are fleeing conflicts, exacerbated by climate change and resource tensions. A watershed moment in global political discourse around climate change and migrants occurred in 2015–2016, when a series of international initiatives acknowledged not only the urgent need to address climate change but also the intersection of climate change and human mobility (see Table 2). In stark contrast to the MDGs, the Sustainable Development Goals (SDGs) called for the “facilitation of orderly, safe, and responsible migration and mobility of people, including through implementation of planned and well-managed migration policies.” The Sendai Framework for Disaster Risk Reduction recognized climate change as a driver of human mobility. The 2017 World Bank Report and the 2019 Atlas of Environmental Migration authored by the IOM and others conclusively recognize the salience of “environmental migrants.”

**Table 2:** A series of international initiatives to address climate change

**COP16 Cancun Adaptation Framework, 2010** The United Nations Framework Convention for Climate Change (UNFCCC) first recognizes the growing importance of human mobility while establishing the Cancun Adaptation Framework at the COP16 in Cancun, Mexico. The conference also established a process for least developed countries (LDCs) and other interested developing countries to formulate and implement national adaptation plans (NAPs) to identify and address their medium and long-term adaptation needs. It proposed the idea of a climate risk insurance facility, and sought “ways to address rehabilitation from the impacts of such climate change-related events as sea-level rise [100].”	
**The Nansen Initiative, 2012** [••^101^] In response to COP16, the Nansen Initiative, launched by Switzerland and Norway, was a state-led consultative process, which recognized that while most movement will take place within countries, there remains a significant protection gap (both legal and operational) for those who cross an international border. Based on these consultations, the Initiative published a consensus “Agenda for the protection of cross-border displaced persons in the context of disasters and climate change,” which was endorsed by 109 states in 2015 [102]. The Protection Agenda supports the integration of effective practices by states and (sub-) regional actors into their own normative frameworks, rather than calling for a new binding international convention on cross-border disaster displacement [103].	
**COP19 Warsaw International Mechanism, 2013** The WIM created a legitimate policy space to discuss and address the negative consequences of climate change if society’s efforts to mitigate and adapt are not sufficient. It sought to implement approaches to address climate change associated loss and damage and recognized migration as an adaptation strategy.	
**COP21 Paris Agreement, 2015** The Paris Agreement seeks to strengthen the global response to the threat of climate change by keeping a global temperature rise this century well below 2 °C above pre-industrial levels. To strengthen the ability of countries to address the impacts of climate change, it seeks to facilitate appropriate financial flows, a new technology framework, and an enhanced capacity-building framework to align action by developing countries and the most vulnerable countries, with their own national objectives. To date, 189 of the 197 Parties to the Convention have ratified it. Having notified intent, the USA can officially withdraw from the Agreement on or after November 4, 2020. Many of the Nationally Determined Contributions, as required by the Paris Agreement, from Africa, Asia Pacific, and Oceania refer to human mobility and its role as an adaptation strategy [9].	
**Sendai Framework for Disaster Risk Reduction, 2015** [••^104^] The Sendai Framework adopted at the World Conference on Disaster Risk Reduction and endorsed by the UNGA in 2015 recognizes that population movements produce risk but can also serve as an adaptation strategy [105]. The text however emphasizes disaster-related displacement and avoids explicit inclusion of mobility from conflict and violence, to gain Member State consensus.	
**Agenda for Humanity, 2016** [106] The third of five principles, articulated in this political communique led by the UN Secretary General, as an output of the World Humanitarian Summit (WHS) in Istanbul, is titled “leave no one behind” and addresses displacement, migration, and statelessness.	
**The Platform on Disaster Displacement, 2015** [107] The goal of the Platform also launched at the WHS is to follow-up on the Protection Agenda published by the Nansen Initiative. It explicitly recognizes the intersection of environment and climate change, and displacement. Its four strategic priorities include addressing knowledge gaps, promoting identified effective practices, promoting policy coherence and mainstreaming of human mobility challenges, and promoting policy and normative development in gap areas [108].	
**New York Declaration for Refugees and Migrants, 2016** [•^109^] The New York Declaration was adopted in the 71st UN General Assembly and addresses migration due to environmental and/or climate change, as well as the environmental impacts of migration, large population movements, and the environmental sustainability aspects of migration. It notes that environmental factors drive both internal and international migration	
**The Global Compact for Safe, Orderly and Regular Migration, 2018** [110] The Compact is an intergovernmental negotiated, non-binding, UN global agreement initially adopted by 160 countries that seeks to address concerns of state sovereignty and responsibility-sharing while upholding human rights and principles of non-discrimination as societies undergo demographic, economic, social, and environmental changes that may have implications for migration or result from it.	
**The Global Compact for Refugees, 2018** [111] The Compact seeks to invest in host communities to provide better education, healthcare access, and livelihood opportunities, moving away from contemporary dominant encampment policies [112].	

There are to date no international conventions that explicitly address migration from environmental or climate-related causes, and no single UN agency responsible for them. Proposals for treaties and legal instruments focusing on climate refugees have largely originated from academia and NGOs and are summarized in Table 3. The IOM does not favor a separate category of “climate refugees” or new treaties to address them, arguing that international attempts at controlling such flows will clash with current notions of national sovereignty [118]. Migration is now recognized not only as a failing consequence of myriad and intersecting determinants, including the failure to adapt to environmental changes, but also as a useful climate-adaptation strategy—if managed, planned, facilitated, and well prepared for in advance [119].

**Table 3:** Key legal instruments that have been proposed in the twenty-first century mostly based on the premise that current options are not sufficient or are failing. The summaries here are based on [113]

**Draft Convention on the International Status of Environmentally Displaced Persons, 2008.** [114] University of Limoges The principles underlying the Convention include the principle of solidarity, common but differentiated responsibilities, effective protection, non-discrimination, and non-refoulement. The specific rights guaranteed to persons threatened by displacement include rights to information and participation, displacement, and the right to refuse displacement. The rights guaranteed to persons already displaced include those common to inter-state and internally displaced persons. The Convention would include a World Fund for the Environmentally Displaced that would provide financial and material assistance for the receipt and return of the environmentally displaced.	
**Proposal for a Convention on Climate Change Refugees, 2009** [115] Harvard University The authors argue for a new, independent convention that allows for the instrument to be creatively tailored to the complexity of the problem and to take a broad-based and integrated approach, on the basis that the problem of climate-induced migration “is sufficiently new and substantial to justify its own legal regime,” instead of being forced within legal frameworks that were not designed to handle it.	
**Proposal for UNFCCC Protocol on the Recognition, Protection and Resettlement of Climate Refugees** [114] Vrije Universiteit Amsterdam Biermann and Boas propose a new Protocol on Recognition, Protection and Resettlement of Climate Refugees under the UNFCCC, since a network of implementing agencies already exist. Rather than create a new convention, this protocol would “provide for the resettlement and reintegration of affected populations over a period of years; offer permanent immigrant status for climate refugees to the regions or countries that accept them; focus on the needs of entire groups of people rather than individuals; provide support for governments, local communities, and national agencies to protect people within their territories; and emphasize the need to protect climate refugees as a global problem and a global responsibility.” The Protocol would be funded by grants to the newly created Climate Refugee Protection and Resettlement Fund.	
**Convention for Persons Displaced by Climate Change, 2010** [116] University of Western Australia The Convention would assign rights and protections through a process of “request and determination” that would be based on scientific studies and the particular situation of the community. Under the Convention, displacement would be viewed as “a form of adaptation that creates particular vulnerabilities requiring protection as well as assistance through international cooperation.” Thus, the emphasis of this Convention would be on the duty of a particular state to provide protection and humanitarian assistance to climate change displaced persons within its jurisdiction and to support governments, local communities, and agencies in fulfilling that duty.	
**Climate Exile Treaty, 2010** [117] IIT, Madras, India The authors distinguish between “Luxury” emissions referring to those associated with wasteful lifestyle choice and “survival” emissions that are associated with subsistence living. They invoke the “beneficiary pays” principle, which states that “countries that undertook and benefited from emissions activities are liable for the costs of combating negative externalities that resulted from them.”	

Most migration will continue to occur within countries, and will largely involve rural to urban population flows, mediated by a complex intersection of political, demographic, socioeconomic, and environmental drivers [120]. While transient shocks like the nationwide lockdown in India amidst the COVID-19 pandemic resulted in return migration to rural areas, we find that the current literature suggests overwhelming net rural to urban migration. The 2011 Foresight Commission Report from the UK government cautioned against trying to prevent such migration (although it might lead to increased vulnerability of new urban migrants) because preventing them from leaving highly stressed or insecure environments could lead to graver outcomes for those trapped in rural areas with even less access to food and water [7]. It recognized that the cities receiving these migrants were also particularly vulnerable to environmental change and without adequate preparation would also suffer the consequences of extreme heat and depleted water resources.

Now, almost a decade later, this scenario is already evident: rural populations moving into vulnerable urban cities, finding no sustenance are on the move again. A recent model developed jointly by the *New York Times Magazine* and *ProPublica*, and published in July 2020, estimates that the number of migrants arriving at the US border from Central America and Mexico may rise to 1.5 million a year by 2050, from about 700,000 a year in 2025, in the absence of climate mitigation or adaptation strategies [121].

The current international agreements are not remotely adequate to address ongoing and projected levels of human migration, regardless of how and how much climate change may influence it, nor are the decades-long and still current trajectories of industrialization, globalization, and development providing any signs of serious commitment to climate adaptation. Realistic global and regional planning should therefore assume the worst-case scenarios under RCP 8.5 and begin immediately to prepare to make life tenable for the hundreds of millions that will urbanize and then attempt to move again as life becomes unendurable when the urban centers themselves exhaust resources and opportunities: the migration in the Dry Corridor heading toward the US border, or the perilous journeys over the Mediterranean, are examples of migration patterns when the first stop of refuge is no longer a sustainable option.

The wall-building, xenophobic, and insular strategy embraced by the USA, Europe, China, and India to deal with the greatest challenge of our times is regressive, violent, and profoundly ignorant, in that it denies the core reality that for millennia, under threat, humans have moved to escape. It dismisses recent and strengthening scientific evidence that stability and security for hundreds of millions in Latin America, Africa, and Asia will depend on the opportunity to relocate, even if seasonally, for work. Facilitating visa, employment, and remittance arrangements can complement urgently needed climate mitigation strategies. Water management in agricultural lands and in urban areas is likely to become critical to any adaptation plan. Host communities, including the transition cities and towns we have examined and final destinations whether in-country or across borders, must start preparing now for the increased demand for food, water, shelter, services, and jobs that will arrive with these migrant populations. There is little indication that any of this preparatory action will happen: the 2020 pandemic has reaffirmed that the current global approach to solving intractable challenges does not embrace cooperation, mutual interest, and scientific rigor but instead retreats to adamant rejection of a future that is upon us.

## Conclusion

The post–WWII international regime sought to rescue, provide refuge, and rehabilitate millions rendered homeless by the wars and post-colonial independence struggles that followed. But in recent decades, large pulses of migration along the dry corridor in Central America, the Sahel, the Middle East, and Asia, some in the context of protracted wars, have culminated in a regime of reneged promises to protect, refusal to let the vulnerable in, and attempts at repatriation or worse, forced refoulement [122]. These responses have followed the ascendancy of inward-looking nation-states in the North and West who have observed explosive population growth elsewhere and interpreted this phenomenon as a threat to their own internal resources and stability. Consequently, international borders have become increasingly tightly regulated and hostile to foreign entrants. Complex visa arrangements are now a key driver of who can cross international borders, and under what conditions, inflicting with their harsh differentiating categories particularly punishing effects on women, children, and the elderly [123].

The regressive treatment of refugees at the US-Mexico border and at the gates of Europe (with the exception of Germany) is diametrically opposite to what is needed. Weaker economies like Turkey, Jordan, Lebanon, and even Bangladesh have been kinder to the millions that have poured across their borders, albeit with wide variation in permitted integration. Unless the most powerful governments around the world change course now—and they are showing little signs of doing so—over this century, the evidence shows, the impact of climate change on the hundreds of millions who will nevertheless move, on those in regions that will receive them, and on those who will not find any remedy through migration, will be impossible to bear.
